# *Rhus coriaria* L. Fruit Extract Prevents UV-A-Induced Genotoxicity and Oxidative Injury in Human Microvascular Endothelial Cells

**DOI:** 10.3390/antiox9040292

**Published:** 2020-04-01

**Authors:** Emma Nozza, Gloria Melzi, Laura Marabini, Marina Marinovich, Stefano Piazza, Saba Khalilpour, Mario Dell’Agli, Enrico Sangiovanni

**Affiliations:** 1 Department of Pharmacological and Biomolecular Sciences (DiSFeB), Università degli Studi di Milano, 20133 Milan, Italy; emma.nozza@studenti.unimi.it (E.N.); gloria.melzi@unimi.it (G.M.); marina.marinovich@unimi.it (M.M.); stefano.piazza@unimi.it (S.P.); enrico.sangiovanni@unimi.it (E.S.); 2 Department of Environmental Science and Policy (ESP), Università degli Studi di Milano, 20133 Milan, Italy; laura.marabini@unimi.it; 3 Boston University School of Medicine, Arthritis Center/Rheumatology, Boston, MA 02118, USA; saba.khalilpour@unimi.it

**Keywords:** *Rhus coriaria* L., sumac, UV-A, antioxidant, genotoxicity, microvascular endothelial cells, HMEC-1

## Abstract

*Rhus coriaria* L. (sumac) is a small plant widely diffused in the Mediterranean region. Its fruit are often consumed as a spice but are also present in traditional medicine of several countries. Recently, interest in this plant has increased and many scientific works reported its beneficial effects including antioxidant and anti-inflammatory properties. Plant extracts can be successfully used against ultraviolet rays, which are able to reach and damage the human skin; however, sumac extracts were never applied to this usage. Thus, in this study, we used a macerated ethanol extract of *Rhus coriaria* L. dried fruit (mERC) to demonstrate its preventive role against the damage induced by ultraviolet-A rays (UV-A) on microvascular endothelial cells (HMEC-1). In vitro effects of the extract pre-treatment and UV-A exposure were evaluated in detail. The antioxidant capacity was assessed by reactive oxygen species (ROS) formation and cellular antioxidant activity measurement. Genoprotective effects of mERC were investigated as well. Our findings indicate that the extract acts as a cell cycle inhibitor or apoptosis inducer, according to the level of damage. The present work provides new insights into the usage of *Rhus coriaria* extracts against skin injuries.

## 1. Introduction

Since ancient times, natural products have been used as remedies for the treatment of several pathological conditions, including skin diseases [1].

*Rhus coriaria* L., commonly called sumac or Sicilian sumac, is a small tree native to southern Europe, belonging to the Anacardiaceae Family. The red fruit of *Rhus coriaria* is used as a very popular spice in Persian countries, either in pure form or in combination with other spices, due to their sour lemon taste. Sumac is traditionally used in Asia and Europe as food or medicinal herb for the treatment of diarrhea, hemorrhoids, and gout [2,3].

Phytochemical studies have shown that fruit are rich in hydrolysable tannins, mostly gallotannins, gallic acid derivatives, anthocyanins, and various organic acids such as malic and citric acids, fatty acids, vitamins, and terpenes [4,5,6,7].

According to the literature, Sumac fruit possess a multitude of biological activities including beneficial effects in vivo in streptozotocin-induced diabetes [8], lipid-lowering effects in hypercholesterolemic rats [9], cardioprotective effects in hyperlipidemic patients [10,11], prevention of necrotizing enterocolitis [12], neuroprotective effects in a mouse model of ischemic optic neuropathy [13,14] and an in vitro model of retinal degeneration [15]. Moreover, Chakraborty A. and colleagues showed in vivo scavenging effects of sumac and inhibition of DNA bases oxidation, also following γ-irradiation [16]. *Rhus coriaria* L. extracts were also used on diabetic patients, where notably decreased serum glucose levels [11]. Recently, our group demonstrated the beneficial effect of *Rhus coriaria* L. fruit extracts as preventive agents in the treatment of keratinocytes inflammation through their inhibition of skin pro-inflammatory mediators, including IL-8, MMP-9, ICAM-1, and VEGF [6].

Botanicals are more and more used in the treatment of skin disorders for their ability to efficiently counteract the damage induced by environmental agents, such as sunlight [17,18,19]. Ultraviolet rays are the most dangerous component of the solar radiation, both UV-A and UV-B are able to act on epidermal cells, damaging them, and UV-A can also penetrate into the dermis [20].

UV-A (320-400 nm) are the most abundant fraction of ultraviolet rays to reach the Earth’s surface due to their ability to pass through the atmosphere, the ozone layer, and through clouds and glass, leading also to a relevant indoor exposure [21]. Since 2009, UV-A rays are included in class I carcinogenic substances from IARC [22]. UV-A exposure can induce both acute and chronic effects: firstly, erythema, immediate pigment darkening (IPD) and persistent pigment darkening (PPD), then, photoaging and carcinogenesis [23].

UV-A rays are responsible for damaging the whole dermic layer, altering collagen, elastin, and activating metalloproteases (MMPs), all leading to tissue photoaging [24,25,26]. Furthermore, their action on fibroblasts and even more on endothelial cells can determinate another negative effect on the epidermis [18,27]. 

At molecular level, UV-A rays mediate the formation of reactive oxygen species (ROS), alter proteins and lipid structures, damage DNA, and promote inflammation processes. UV-A induced damage is mainly oxidative, and this is particularly relevant in endothelial cells, whose alteration could compromise the whole function of vessels [28].

To correctly mimic the complex situation of dermal vessels in vitro, an immortalized human microvascular endothelial cell line (HMEC-1) was chosen; these cells are commonly used as in vitro model of the skin microvascular endothelium [29]. The reliability of this model as a tool for the study of UV-A irradiation has been previously reported [30].

This work investigates the photoprotective effect of a macerated ethanolic extract of *Rhus coriaria* L. dried fruit (mERC) against UV-A damage and the genoprotective effects in microvascular endothelial cells. Damage and protection were examined through evaluation of the effects on oxidation, genotoxicity, and cytotoxicity.

## 2. Materials and Methods

### 2.1. Rhus coriaria L. Extract Preparation and Characterization

*Rhus coriaria* fruit were purchased in the Taleghan region (Iran), and plant material was authenticated by the Herbarium Unit, School of Biological Sciences. For extract preparation, dried fruit was ground, and 5 g was extracted in pure ethanol (50 mL) at room temperature (RT) for 48 h under stirring, as previously reported [6]. The mixture was filtered, taken to dryness, and freeze dried, obtaining *Rhus coriaria* L. macerated extract (mERC). The extraction yield was 15.2%. Phytochemical characterization by HPLC-UV-DAD analysis showed the presence of gallotannin derivatives and flavonoids, including anthocyanins (cyanidin derivatives), as reported in [6].

### 2.2. Cell Culture

The HMEC-1 cell line (Centers for Disease Control and Prevention, Atlanta, GA, USA) was kindly gifted by Prof. Nicoletta Basilico, Dipartimento di Scienze Biomediche, Chirurgiche e Odontoiatriche, Università degli Studi di Milano (Italy). Cells were grown in MCDB 131 medium (Sigma Aldrich) plus 1% penicillin/streptomycin, 10% fetal bovine serum (FBS), 20 mM HEPES buffer, 1 μg/mL hydrocortisone and 10 ng/mL epidermal growth factor (EGF) [31]. Cells were maintained at 37 °C in a 5% CO_2_ atmosphere and passaged every 3/4 days. For experiments, where not differently stated, cells’ growth lasted 48 h.

### 2.3. Rhus coriaria L. Extract Treatment

Cells were incubated for 1 h with mERC diluted in a serum-free medium at the final concentrations of 10 or 25 μg/mL (E10, E25). The incubation was performed right before UV-A exposure. 

### 2.4. UV-A Radiation Treatment

UV-A treatments were performed using a four-lamp UV-A system (TRIWOOD 31/36, Helios Italquartz). During UV-A exposure, cells were maintained in a thin layer of phosphate buffered saline (PBS; 2 mL in 60 mm Petri dishes, 1 mL in 35 mm Petri dishes, 500 μL in 24-well plates, 100 μL in 96-well plates) and their supports encircled by ice, to avoid external interferences [32]. Moreover, control samples were maintained in PBS at RT for the same time. In this study, four different UV-A doses, 10, 15, 20, and 25 J/cm^2^ (T10, T15, T20, T25) were tested. Unless different indications, experiments were performed directly after UV-A treatment. 

### 2.5. ROS Quantification and Lowry Protein Assay

To evaluate the production of intracellular ROS, a fluorescence spectrometry protocol combined with Lowry protein quantification was applied. 1x10^4^ cells were seeded on a 96-well black plate; after treatment, cells were diluted 1:2000 in medium and incubated for 30 min with 50 mM fluorescent probe (DCFH-DA, 2′,7′-dichlorofluorescein diacetate, Sigma Aldrich). The probe binds cytoplasmatic ROS and emits fluorescence (Figure S1), which is detected with a spectrofluorometer at 495 nm wavelength. Fluorescence values were normalized on the protein content of each sample, after the quantification with Lowry protocol [33], to obtain FU/μg proteins (fluorescence units on micrograms of proteins).

### 2.6. Quantification of the Total Antioxidant Activity

The total antioxidant activity was evaluated using a colorimetric reaction kit (MAK 187, Sigma, Milan, Italy). The assay was performed following the manufacturer’s instructions on cell lysate. Briefly, HMEC-1 were seeded on 60 mm Petri dishes at a density of 2.15 × 10^4^ cell/cm^2^ and grown for 72 h, before treatment. Then, cells were scraped, collected, and mechanically lysed using a potter. Absorbance values were compared with the Trolox standard curve and expressed as Trolox equivalents normalized on cell viability (MTT).

### 2.7. Alkaline Comet Assay

The alkaline Comet assay is a well-known single cell gel electrophoresis (SCGE) protocol for evaluating DNA damage. It was performed immediately or 24 h after treatment, on cells seeded on 35 mm Petri dishes (1.5 × 10^4^ cell/cm^2^ density). To perform the experiments, cells were detached using a trypsin-EDTA solution (0.05%, Sigma Aldrich) and seeded on agarose-coated slides; to create an “agarose sandwich” another layer was spread and left to cool. Later, slides were soaked in a lysis solution (10% DMSO, 1% Triton X-100, 89% Stock solution: 2.5 M NaCl, 250 mM NaOH, 100 mM Na_2_EDTA, 10 mM Tris in water; Sigma Aldrich) for 30 min at 4°C. Subsequently, slides were neutralized (0.4 M Tris in water) and kept for 30 min in stabilization inside an electrophoresis system with an alkaline buffer (300 mM NaOH, 1 mM Na_2_EDTA in water), for DNA unwinding. At the end of the stabilization, the electrophoretic run was performed for 30 min, at 300 mA and 25V. Slides were neutralized again and colored with propidium iodide to be read in fluorescence microscopy (40× oil obj., Figure S2). An amount of 100 cells/sample was analyzed using software (TriTek CometScore™). 

### 2.8. Modified Comet Assay

A modified Comet assay protocol was used to identify the different kind of DNA damage [34]. The alkaline Comet assay was performed with some variations: the pH of the alkaline buffer was set at 12.1; after the lysis, slides were incubated for 45 min at 37 °C with 50 μL of the diluted enzymes: T4 PDG, ENDO III, FPG (New England BioLabs^®^ Inc., 75-77 Knowl Piece, Wilbury Wai, Hitchin, UK). Enzymes were diluted in T4 PDG Reaction buffer to reach final concentrations of 10 U/mL for T4 PDG and FPG, 14 U/mL for ENDO III, according to manufacturer’s instructions.

### 2.9. Quantification of γ-H2AX and Micronuclei Percentage

The evaluation of DNA double strand break (DSB) was conducted through immunofluorescence, quantifying the phosphorylated form of the H2AX histone (γ-H2AX). 3x10^4^ cells/cm^2^ were seeded on round glass slides (12mm). After treatment, cells were fixed with iced methanol and washed with PBS, then incubated, first with a permeabilizing solution (0.5% Triton X-100 in PBS), and later with a 3% PBS/BSA solution for 1 h. Subsequently, cells were incubated overnight at 4°C with a primary antibody (Histone H2AX.XS139ph antibody, Active Motif) that binds the Ser139 residue on the γ-H2AX histone. The day after, slides were washed and incubated for one hour with the secondary antibody (AlexaFluor 488 Goat anti-rabbit IgG H+L, Immunological Sciences). Antibodies were diluted according to the manufacturer’s instructions. Then, cells were washed again, and slides mounted using 10 μL of DAPI (VectaShield, Vector). The reading was performed in fluorescence microscopy (100X oil obj.) using FITC and DAPI filters; 100 cells/sample were analysed and divided into three classes of damage: 0–5, 6–10, >10 *foci*. From the same samples was possible to count 1000 cells each and identify micronuclei, following Fenech’s criteria [35]. 

### 2.10. Evaluation of Apoptosis

To evaluate the percentage of dead cells a flow cytometry protocol based on Annexin V property of binding exposed phosphatidylserine (PS) in apoptotic cells was followed; propidium iodide was used to bind necrotic cells’ DNA. 3 × 10^4^ cells/cm^2^ were seeded on 35 mm Petri dishes; after treatment, cells were incubated in serum-free medium for 24 h. At the right time, cells were detached using a trypsin-EDTA solution (0.05%) and incubated for 15 min at RT with both Annexin V and propidium iodide, diluted in Annexin Binding Buffer (1:20 Annexin V, 1:1000 propidium iodide; ThermoFisher). After incubation, samples were read on flow cytometry, analyzing 10 000 cells each (Figure S3). 

### 2.11. Cell Cycle Analysis

Cell cycle analysis was conducted in flow cytometry, measuring the DNA quantity of each cell that corresponds to a different phase of the cycle. To perform the test, 3 × 10^4^ cells/cm^2^ were seeded on 35 mm Petri dishes, treated, and incubated in serum-free medium for 24 h. Cells were then detached using a trypsin-EDTA solution (0.05%), collected and fixed with absolute ethanol overnight at 4°C. Afterwards, cells were incubated for 40 min with a staining solution (0.1% Triton X-100, 5% propidium iodide, 0.5% RNase A in PBS; Sigma Aldrich). To perform the analysis (20 000 cells/sample), the solution was removed, and cells resuspended in PBS. 

### 2.12. Statistical Analysis

For conducting the statistical analysis, the samples exposed only to PBS were used as controls; samples treated only with the extract gave results similar to untreated controls for every assay.

The statistical analysis of the experimental results was performed using the statistical software GraphPad Prism 5.01 (GraphPad Software), through the One-Way ANOVA test, associated with Bonferroni’s Multiple Comparison Test. For all the experiments at least three separated data were collected and shown in the graphs as mean ± SEM; for the two Comet assays the mean of medians ± SEM was shown. 

## 3. Results

### 3.1. Rhus coriaria L. Extract Decreases UV-A-induced ROS Production 

One of the most common mechanisms increased by UV-A exposure is the production of intracellular ROS, which causes damage to the main cellular structures, also comprising DNA [36]. Endothelial cells are sensible to ROS-induced injury, which is one of the mechanisms responsible for endothelial dysfunction [37]. 

**Figure 1:**
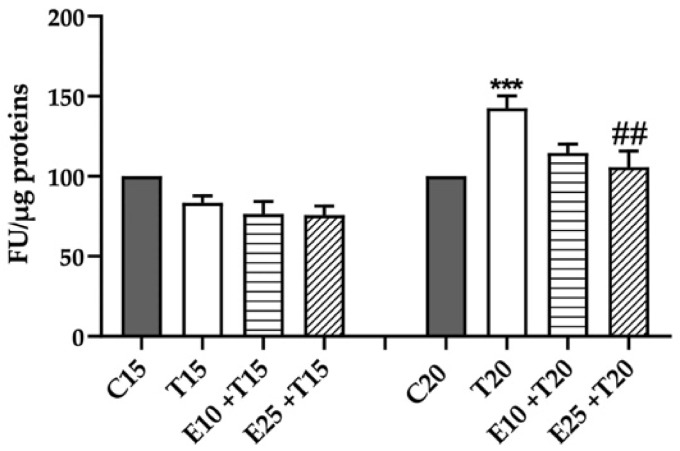
ROS production, expressed as Fluorescence Units on protein content (μg), after mERC and UV-A treatment. HMEC-1 were treated for 1 h with 10 or 25 μg/mL of mERC (E10, E25) and exposed to 15 or 20 J/cm^2^ UV-A (T15, T20). Results are expressed as mean ± SEM, *n* = 5. Statistical analysis: One-Way ANOVA with Bonferroni’s post hoc analysis. *** *p* < 0.001 vs. C20, ## *p* < 0.05 vs. T20.

As shown in Figure 1, the higher dose of UV-A tested (20 J/cm^2^) induced a great intracellular ROS production, which was prevented by the antioxidant properties of mERC pre-treatment. mERC (25 μg/mL) was able to decrease ROS production to control levels.

### 3.2. Total Antioxidant Activity Reduction after UV-A Exposure in the presence of mERC

Endogenous antioxidant activity plays a major role in contrasting reactive species formation; to maintain the redox homeostasis various enzymes as superoxide dismutase (SOD) or catalase (CAT) and non-enzymatic compounds are involved [38]. 

**Figure 2:**
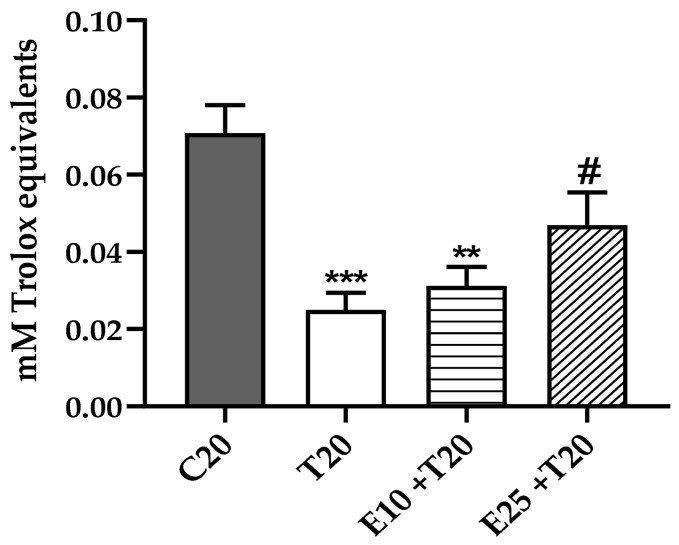
Effect of *Rhus coriaria* L. extract and UV-A rays on total cellular antioxidant activity, measured as mM Trolox equivalents after normalization on cell’s viability. HMEC-1 were treated for 1 h with 10 or 25 μg/mL of mERC (E10, E25) and exposed to 20 J/cm^2^ UV-A (T20). Results are expressed as mean ± SEM, *n* = 5. Statistical analysis: One-Way ANOVA with Bonferroni’s post hoc analysis. ** *p* < 0.05 *** *p* < 0.001 vs. C20, # *p* < 0.01 vs. T20.

UV radiation can increase ROS formation and consequently diminish the antioxidant capacity of cellular systems. Indeed, cellular antioxidant activity was measured only at 20 J/cm^2^ UV-A, where high release of ROS was detected (Figure 1), and the values normalized on cells’ viability (Table S1). The total cellular antioxidant activity was significantly decreased in UV-A treated samples (Figure 2).

The antioxidant ability of *Rhus coriaria* L. extracts was partially documented [39,40]; however, this study shows for the first time in endothelial cells the efficacy of mERC to counteract oxidative stress at low concentration (25 μg/mL, Figure 2). 

### 3.3. Genoprotective Action of mERC Against UV-A Damage

The beneficial effects of *Rhus coriaria* L. extracts presented in the literature are mainly focused on their antioxidant properties, and little is known about their genoprotective potential. 

UV-A radiation has to be considered a remarkable DNA damage inducer that could be prevented by mERC pre-treatment.

#### 3.3.1. Evaluation of DNA Damage Through Alkaline Comet Assay

Alkaline Comet assay highlights the presence of different kind of DNA damages, as single strand break (SSB), double strand break (DSB) or alkali-labile sites (ALS), measuring DNA fragmentation through different parameters: % DNA in Tail, Tail length and most importantly Tail moment, which correlates with the other two parameters.

**Figure 3:**
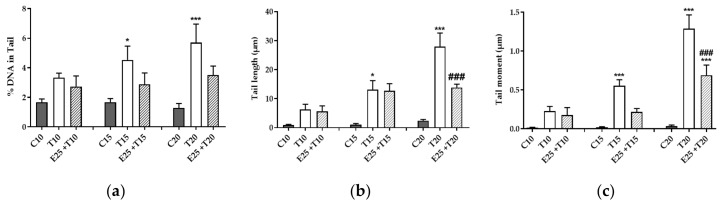
Effect of *Rhus coriaria* L. and UV-A rays on genotoxicity. Alkaline Comet assay evaluated DNA damage through (**a**) %DNA in Tail, (**b**) Tail length and (**c**) Tail moment (%DNA in Tail* Tail length). HMEC-1 cells were treated for 1 h with 25 μg/mL of mERC (E25) and exposed to 10, 15 or 20 J/cm^2^ UV-A (T10, T15, T20). Results are expressed as mean of medians ± SEM, *n* = 6. Statistical analysis: One-Way ANOVA with Bonferroni’s post hoc analysis. * *p* < 0.05 *** *p* < 0.001 vs. C PBS, ### *p* < 0.001 vs. T20.

All parameters considered in the Comet assay (Figure 3) showed a significant increase of DNA damage after UV-A exposure, which was genotoxic in endothelial cells at 15 and 20 J/cm^2^. 

mERC’s pre-treatment allowed a decreasing trend of genotoxicity at all UV-A doses, but a significant damage reduction was observable only at 20 J/cm^2^ of UV-A irradiation, especially on Tail length and Tail moment parameters (Figure 3b,c). The extract was effective only at its higher concentration whereas, at 10 μg/mL, no differences from UV treated samples were showed (data not shown). 

#### 3.3.2. Time Course of Genotoxic Damage

The damage on DNA induced by UV-A is remarkable when detected shortly after UV-A exposition, as discussed above (Figure 3), but after 24 h can be reduced by the activation of cellular repair enzymes [41] or by the elimination of the most damaged cells through apoptosis [42]. 

**Figure 4:**
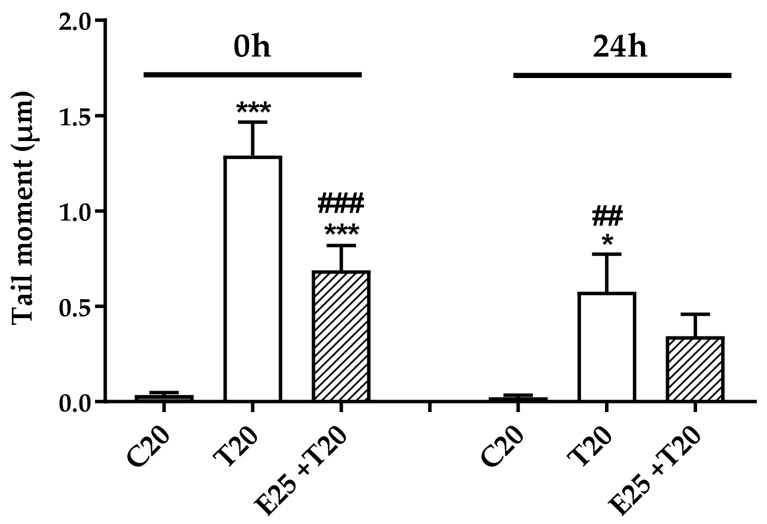
Comparison between Alkaline Comet assay performed after 0 and 24 h from UV-A exposure, expressed as Tail moment. HMEC-1 cells were treated for 1 h with the extract (25 μg/mL, E25) and exposed to 20 J/cm^2^ UV-A (T20). Results are expressed as mean of medians ± SEM, *n* = 3. Statistical analysis: One-Way ANOVA with Bonferroni’s post hoc analysis.* *p* < 0.05 *** *p* < 0.001 vs. C20, ## *p* < 0.01 ### *p* < 0.001 vs. T20 0h.

Figure 4 shows a comparison between the most relevant parameter, Tail moment, evaluated immediately (0 h) or after 24 h from the UV-A exposure; 20 J/cm^2^ was chosen as a representative dose. Indeed, the value of Tail moment after 24 h was significantly decreased when compared to time zero (0 h) (Figure 4).

#### 3.3.3. DSB Quantification through γ-H2AX Detection

To investigate the genotoxic damage induced by UV-A and the action of mERC, the presence of the phosphorylated form of the H2AX histone, which is indicative of an early DSB repair pathway stage [43], was tested. γ-H2AX are visible as *foci*, using an immunofluorescence technique, and their number in the nucleus is indicative of the damage state of the cell. 

**Figure 5:**
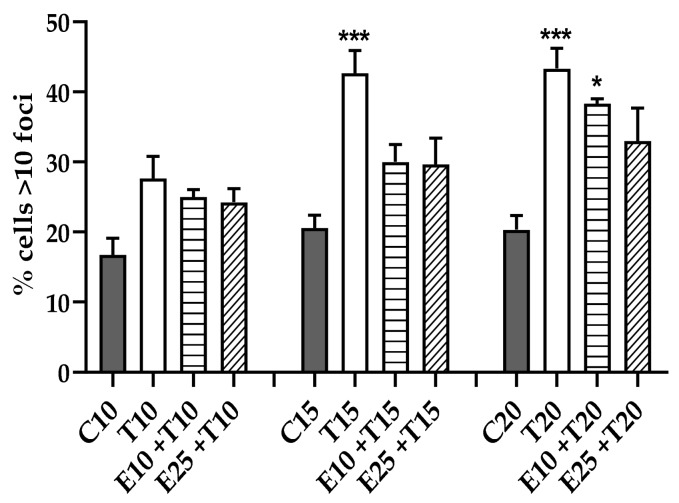
γ-H2AX formation after UV-A irradiation and mERC pre-treatment. DNA DSB quantification through immunofluorescence revealing high (>10) γ-H2AX *foci* presence. HMEC-1 cells were treated for 1 h with mERC (10 or 25 μg/mL, E10 and E25) and exposed to 10, 15, 20 J/cm^2^ UV-A (T10, T15, T20). Results are expressed as mean ± SEM, *n* = 4. Statistical analysis: One-Way ANOVA with Bonferroni’s post hoc analysis. * *p* < 0.05 *** *p* < 0.001 vs. C PBS.

Cells whose nuclei contain more than 10 *foci*, as displayed in Figure 5, are considered heavily damaged [43]. The percentage of these cells in the whole population was significantly increased in UV-A-treated samples, from the dose of 15 J/cm^2^. 

Against this kind of damage, the extract pre-treatment resulted in a decreased number of damaged cells in the population (Figure 5). This result could be explained by the involvement of the γ-H2AX in the mitotic process; indeed, γ-H2AX formation in mitosis is physiological and unrelated from DNA damage [43]. In this assay, cells of each phase of the cycle were considered, making impossible to discriminate either the mitotic presence of the histone or the related damage. 

#### 3.3.4. Chromosomal Mis-segregation as Micronuclei Formation 

The incorrect separation of chromosomes during mitosis is considered a reliable index of genomic damages [44]. This process happens to whole chromosomes or just some parts that do not migrate along with the rest of the spindle and are included in a separate membrane, originating the so-called micronuclei. Through immunofluorescence, using Fenech’s criteria [35], micronuclei can be efficiently recognized, and the state of cellular DNA damage established. 

UV-A irradiation induced a sharp increase of micronuclei percentage in HMEC-1 cells; this rise was significant compared to control samples, from the minimum UV dose tested (10 J/cm^2^). 

**Figure 6:**
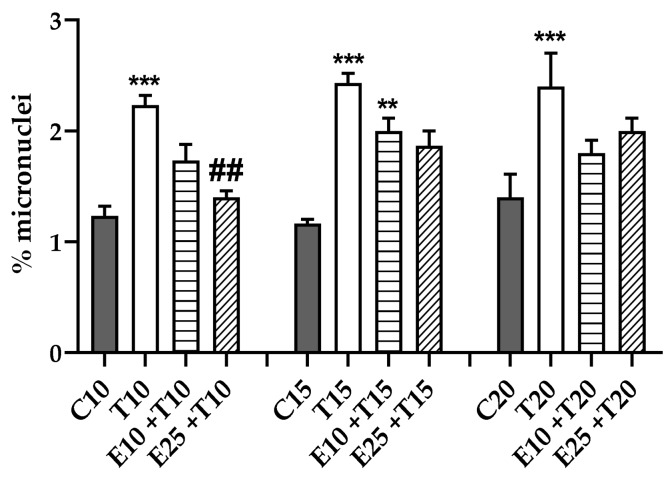
Effect of mERC and UV-A radiation on chromosome mis-segregation, measured as micronuclei percentage, detected by immunofluorescence. HMEC-1 cells were treated for 1 h with mERC (10 or 25 μg/mL, E10 and E25) and exposed to 10, 15, 20 J/cm^2^ UV-A (T10, T15, T20). Results are expressed as mean ± SEM, *n* = 3. Statistical analysis: One-Way ANOVA with Bonferroni’s post hoc analysis. ** *p* < 0.01 *** *p* < 0.001 vs. C PBS, ## *p* < 0.01 vs. T10.

Pre-treatment with mERC decreased the level of damage at every dose of exposition, compared to UV-only treated samples, but the reduction was significant only at 10 J/cm^2^ with the highest extract concentration (Figure 6).

### 3.4. UV-A Damage Characterization

To better investigate the mechanism of action of mERC against UV-A damage the main features of UV-A genotoxicity needed to be investigated. UV-A rays have been considered just inducers of oxidative damage, for their low energy and little capability of being directly absorbed by DNA [36], but recent studies demonstrated that they can also generate direct DNA damages as cyclobutane pyrimidine dimers (CPDs) [45,46,47]. This information provides new perspectives on UV-A damage, since the production of CPDs induces cytokines release, but also a great mutagenicity [48].

The modified Comet assay exploits specific endonucleases to recognize different kind of DNA lesions. Indirect damage is due to bases’ oxidation, ENDO III identifies oxidized pyrimidines and FPG detects oxidized purines. Instead, T4 PDG is able to recognize CPDs, index of direct genotoxicity. 

**Figure 7:**
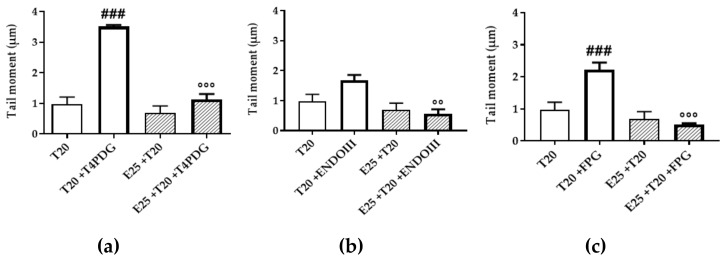
Characterization of genotoxic damage induced by UV-A, and mERC extract’s role. Modified Comet assay measured direct DNA damage using (**a**) T4 PDG enzyme recognizing CPDs; indirect i.e., oxidative damage was identified by (**b**) ENDO III for oxidized pyrimidines or (**c**) FPG for oxidized purines. HMEC-1 cells were treated for 1 h with mERC (25 μg/mL, E25) and exposed to 20 J/cm^2^ UV-A (T20). Results are expressed as mean of medians ± SEM, *n* = 3. Statistical analysis: One-Way ANOVA with Bonferroni’s post hoc analysis. ### *p* < 0.001 vs. T20, °° *p* < 0.01 °°° *p* < 0.001 vs. T20+enzyme.

Figure 7 displays the amount of damage revealed by each endonuclease; they are directly correlated with the incidence of that specific lesion in the overall damage induced by UV-A on HMEC-1 cells. 20 J/cm^2^ was chosen as a representative dose of genotoxicity. In Figure 7a is shown the presence of direct damage, using T4 PDG that appeared significant and resulted the main component of UV-A induced damage. Oxidative damage is visible with ENDO III and FPG, in Figure 7b,c; the most prevalent indirect lesion was the oxidation of purinic bases, while pyrimidines’ oxidation was almost non-detectable.

The effect of mERC’s higher concentration was relevant on both direct and indirect DNA damages (Figure 7); the extract ability to significantly prevent not only oxidative, but also direct lesions is a new outcome.

### 3.5. Assessment of Cytotoxicity

In the previous experiments, the great mutagenic capacity of UV-A rays in endothelial cells was clearly shown; therefore, the accumulation of subsequent damages could lead to mutations that finally result in tumor transformation. One of the main strategies to solve this problem is the activation of the apoptosis pathways.

**Figure 8:**
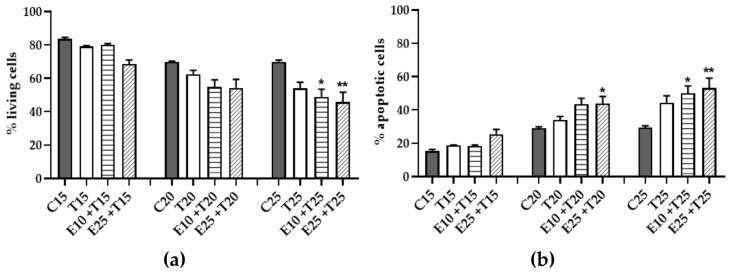
Cytotoxicity of UV-A rays and effects of *Rhus coriaria* pre-treatment, flow cytometry evaluation. Cellular population was grouped in (**a**) living cells and (**b**) apoptotic cells. HMEC-1 cells were treated for 1 h with 10 or 25 μg/mL of mERC (E10, E25) and exposed to 15, 20, 25 J/cm^2^ UV-A (T15, T 20, T25). Results are expressed as mean ± SEM, *n* = 3. Statistical analysis: One-Way ANOVA with Bonferroni’s post hoc analysis. * *p* < 0.05, ** *p* < 0.01 vs. C PBS.

Through flow cytometry, cellular population could be grouped in living or apoptotic cells (Figure S3). In Figure 8a, the living population shows a decreasing trend, correlated with the increase of UV-A exposition, but never significant for UV-only treated samples. The decrement was more accentuated by the extract pre-treatment, resulting in a significant decrease of living cells in samples treated with the maximum UV-A dose (25 J/cm^2^). Figure 8b displays the increase of apoptotic cells, resulting complementary to the living cells; apoptotic population was significantly increased only in mERC pre-treated samples, treated with high UV-A doses (20–25 J/cm^2^).

The increase of apoptotic cells should be read as a protective mechanism by mERC’s pre-treatment against the high levels of cellular damage, to avoid a possible tumor transformation. This feature of polyphenolic extracts was already stated [49]. 

### 3.6. Role of mERC Pre-treatment and UV-A Exposure on Cell Cycle

The induction of apoptosis is one among a variety of mechanisms implemented by cells to avoid the onset of mutations; a sudden repair pathway is the cell cycle arrest that results particularly preferred in CPDs damaged cells [50]. Flow cytometry allows evaluating both.

**Figure 9:**
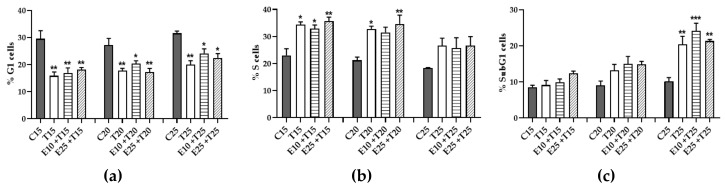
Flow cytometry cell cycle analysis after *Rhus coriaria* and UV-A treatment. Cellular population was grouped in five phases, comprising (**a**) G1 phase, (**b**) S phase, (**c**) SubG1 phase. HMEC-1 cells were treated for 1 h with 10 or 25 μg/mL of mERC (E10, E25) and exposed to 15, 20, 25 J/cm^2^ UV-A (T15, T20, T25). Results are expressed as mean ± SEM, *n* = 3. Statistical analysis: One-Way ANOVA with Bonferroni’s post hoc analysis. * *p* < 0.05, ** *p* < 0.01, *** *p* < 0.001 vs. C PBS.

In Figure 9 the three most relevant phases are shown: G1, S and SubG1. G1 and S phase displayed opposed trends, relevant for the lower UV doses (15-20 J/cm^2^): a decrease in percentage of G1 cells and an increase of S cells. This happened as well in mERC pre-treated samples, indicating cell cycle arrest.

SubG1 phase (Figure 9c) is index of apoptosis and a significant difference from control samples was observable only at the maximum UV-A dose (25 J/cm^2^), where there was a notable increase of the cell’s percentage; even in this condition, pre-treatment with mERC gave a similar outcome to UV-only treated samples, increasing the apoptotic population. This result confirmed the previous hypothesis of the apoptotic pathway as a protective strategy against tumor transformation.

## 4. Discussion

Natural polyphenols exert their protective effects acting on various damage pathways; their main beneficial activity is the antioxidant and radical scavenger ability, but also anti-inflammatory and immunomodulatory properties are relevant [18]. Among the botanicals studied in recent literature, *Rhus coriaria* L. has shown antimicrobial, antiviral, cardioprotective and antihyperglycemic abilities [2] and some extracts displayed also anti-genotoxic features [51].

The ethanolic extract of *Rhus coriaria* L. used in the present work is particularly rich in phenolic acids, including flavonoids and gallotannins. The extract composition was already reported, and showed a peculiar behavior in reducing inflammation in keratinocytes, indicating a possible application in the treatment of skin disorders [6].

Knowing the main properties of *Rhus coriaria* L., it is logical to apply the extract in prevention of skin damages induced by UV-A rays’ exposure. Indeed, UV-A radiation is able to reach all the layers of the skin and penetrate to the deep dermis where it interacts with fibroblasts and also with the microvascular endothelium [18,52].

UV-A rays are able to generate a great amount of intracellular free radicals leading to oxidative stress and cellular damage [28,53]; this is a particularly severe condition for endothelial cells, whose high damage to cellular biomolecules leads to endothelial dysfunction [37]. As shown in this study, after UV-A exposure, the endogenous antioxidant activity is lowered, as a result of the high amount of ROS generation; the extract pre-treatment decreases the formation of these reactive species, while it seems to preserve the antioxidant enzymes and/or their activity. This antiradical activity of *Rhus* extracts was also confirmed in other cell lines, as erythrocytes exposed to pro-oxidant agents [54]. The molecular mechanisms underlying the antioxidant effects need further investigation; however, pure compounds occurring in sumac including gallic acid [55], anthocyanins and other polyphenols are Nrf-2 modulators [56]. Moreover, another species of the same genus (*Rhus verniciflua* Stokes) was shown to induce Nrf-2 activity thus corroborating the hypothesis that *Rhus coriaria* may modulate this transcription factor [57].

UV-A exposure damages the DNA, as demonstrated in this study by various assays, leading to single or double strand breaks. DNA lesions can be originated directly or not, according to different mechanisms: direct damage is due to the interaction between the radiation and the DNA double helix [58], while the induction of photosensitization reactions leads indirectly to DNA detriment [59]. The literature mainly refers to UV-A as an indirect DNA damage source, because the oxidative stress generated is able to affect the main cellular structures, including DNA [58,60]. Instead, this study demonstrates that UV-A exposure leads to a direct injury of the genetic material through the formation of CPDs that are considered to be mainly responsible for UV-B induced mutagenicity [61]. Moreover, UV-A induced also mis-segregation mechanisms, resulting in micronuclei formation. *Rhus coriaria* L. pre-treatment blocked the formation of DNA lesions, confirming a genoprotective effect which was investigated, at least in part, in animal and human models [16]. Thus, our findings suggest that *Rhus coriaria* may exert genoprotective effects against low UV-A exposure through antioxidant-independent mechanisms.

The mode of action of the extract used in the present study needs further investigation; however, our suggestions indicate that the extract retains either antioxidant or radical scavenger ability, which could reduce the indirect damage originated by ROS, or promote the DNA protective capacity. A possible explanation of the extract mode of action is the involvement of a direct filter action against UV-A rays due to the presence of polyphenols which are efficiently up-taken by the cells during the pre-treatment and retained in the cytoplasm before irradiation. Regarding this specific physical photoprotection, it was previously demonstrated that anthocyanins-rich extract from strawberries may protect dermal fibroblasts by UV-A filtration [62].

The production of CPDs implies that UV-A exposure can ultimately lead to tumor transformation. To avoid this dangerous outcome, cells can sense the presence of pyrimidine dimers and induce the arrest of cell cycle [50], as seen at lower UV-A doses, where an intraS phase block occurred to allow the DNA damage repair. Conversely, at the higher UVA dosage tested, the damage resulted was too heavy; in this situation, to avoid tumor proliferation, cells prefer to engage a process that blocks RNA transcription and consequently activates p53 and the apoptotic pathway [63]. Interestingly, *Rhus coriaria* L. extract displayed two opposite behaviors: is genoprotective in cells exposed to medium UV-A doses, while results pro-apoptotic in highly damaged cells, displayed by the accumulation of cells in subG1 phase. The peculiar effect of *Rhus coriaria* L. extract in activating the apoptotic machinery was already observed in another study, where the extract showed anticlastogenic properties [51], a feature reported also for other polyphenolic extracts [49,64,65]. Polyphenol-rich extracts from *Rubus* spp. and grapevine (*Vitis vinifera* L.) exhibit pro-apoptotic effect after UVR exposure in vitro and in vivo, as reported in the literature [66,67,68]. More studies are needed to clarify the mechanism of apoptosis’ induction from the extract pre-treatment along with UV-A induced damage.

## 5. Conclusions

In conclusion, this work highlights the beneficial effects of *Rhus coriaria* L. as a novel source of phytochemicals with antioxidant and antiproliferative properties, while the anti-inflammatorypotential of the extract is still to be investigated. It is noteworthy that the preventive role on microvascular endothelium, against the damage induced by ultraviolet radiation, was exerted at low concentrations (10–25 μg/mL), enabling future applications as local treatment or as dietary supplement or functional food. Knowing the latest technologies in polyphenol vehiculation, a drug delivery approach using nano-carriers [69] for reaching the skin endothelial cells may be useful and worthy of further studies.

## Supplementary Materials

The following are available online at https://www.mdpi.com/2076-3921/9/4/292/s1, Table S1: Cellular viability used for normalization of cellular antioxidant activity values, measured through MTT assay. Figure S1: Intracellular ROS presence: (a) C20, (b) T20, (c) E25+T20. Fluorescence microscopy, obj. 100X oil, DAPI staining for cellular nuclei, FITC staining for ROS. Figure S2: Alkaline Comet assay: (a) C20, (b) T20, (c) E25+T20. Fluorescence microscopy, obj. 40X oil, propidium iodide staining. Figure S3: FACS report of cellular population in Annexin V assay. (a) C25, (b) T25, (c) E25+T25. For each image: Q3-1 represents the necrotic population, Q3-2 and Q3-4 represent the apoptotic population, Q3-3 represents the living population.
