# Supplementary material for: Rhus coriaria L. Fruit Extract Prevents UV-A-Induced Genotoxicity and Oxidative Injury in Human Microvascular Endothelial Cells
Source: Antioxidants (Basel). 2020 Apr 1;9(4):292. doi: 10.3390/antiox9040292 (PMC7222194; doi:10.3390/antiox9040292)
Supplement: Supplementary file 1 [file antioxidants-09-00292-s001.zip › antioxidants-763767-supplementary.docx]

*Rhus coriaria* L. fruit extract prevents UV-A-induced genotoxicity and oxidative injury in human microvascular endothelial cells

Emma Nozza, Gloria Melzi, Laura Marabini, Marina Marinovich, Stefano Piazza, Saba Khalilpour, Mario Dell’Agli, and Enrico Sangiovanni

**Table S1.** Cellular viability used for normalization of cellular antioxidant activity values, measured through MTT assay.

| **Sample** | **% Viability on C20** | **SEM** |
| --- | --- | --- |
| C20 | 100 | ± 0 |
| T20 | 80.5 | ± 4.4 |
| E10 +T20 | 64 | ± 7.6 |
| E25 +T20 | 65.75 | ± 6.7 |

**Figure S1.** Intracellular ROS presence: (**a**) C20, (**b**) T20, (**c**) E25+T20. Fluorescence microscopy, obj. 100X oil, DAPI staining for cellular nuclei, FITC staining for ROS.


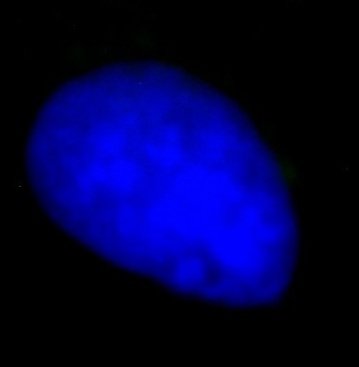

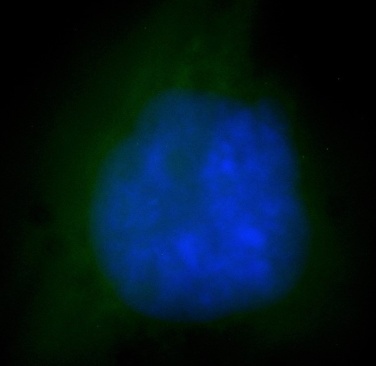

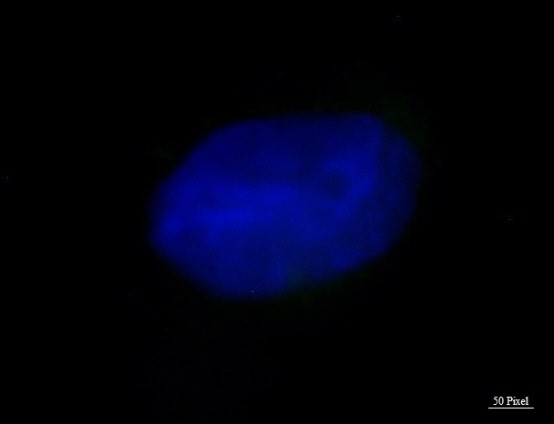


(**a**) (**b**) (**c**)

**Figure S2.** Alkaline Comet assay: (**a**) C20, (**b**) T20, (**c**) E25+T20. Fluorescence microscopy, obj. 40X oil, propidium iodide staining.


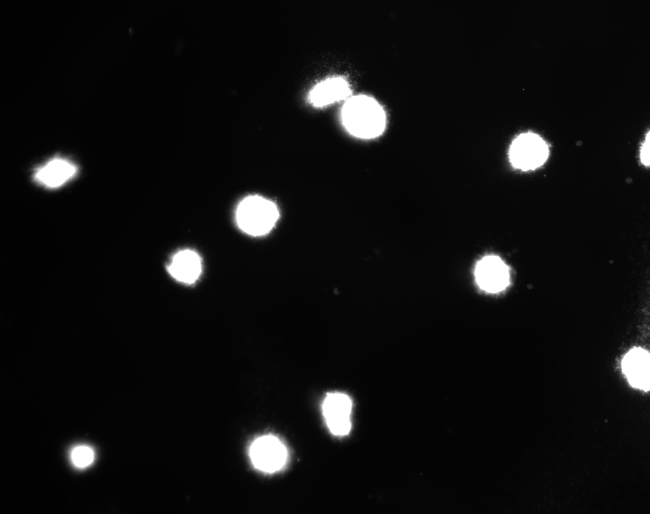

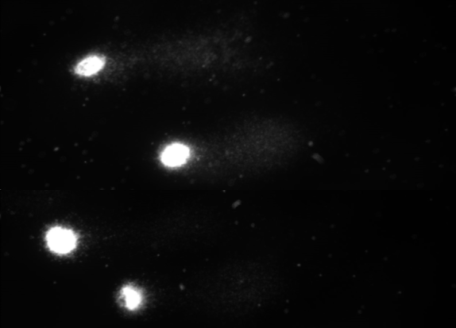

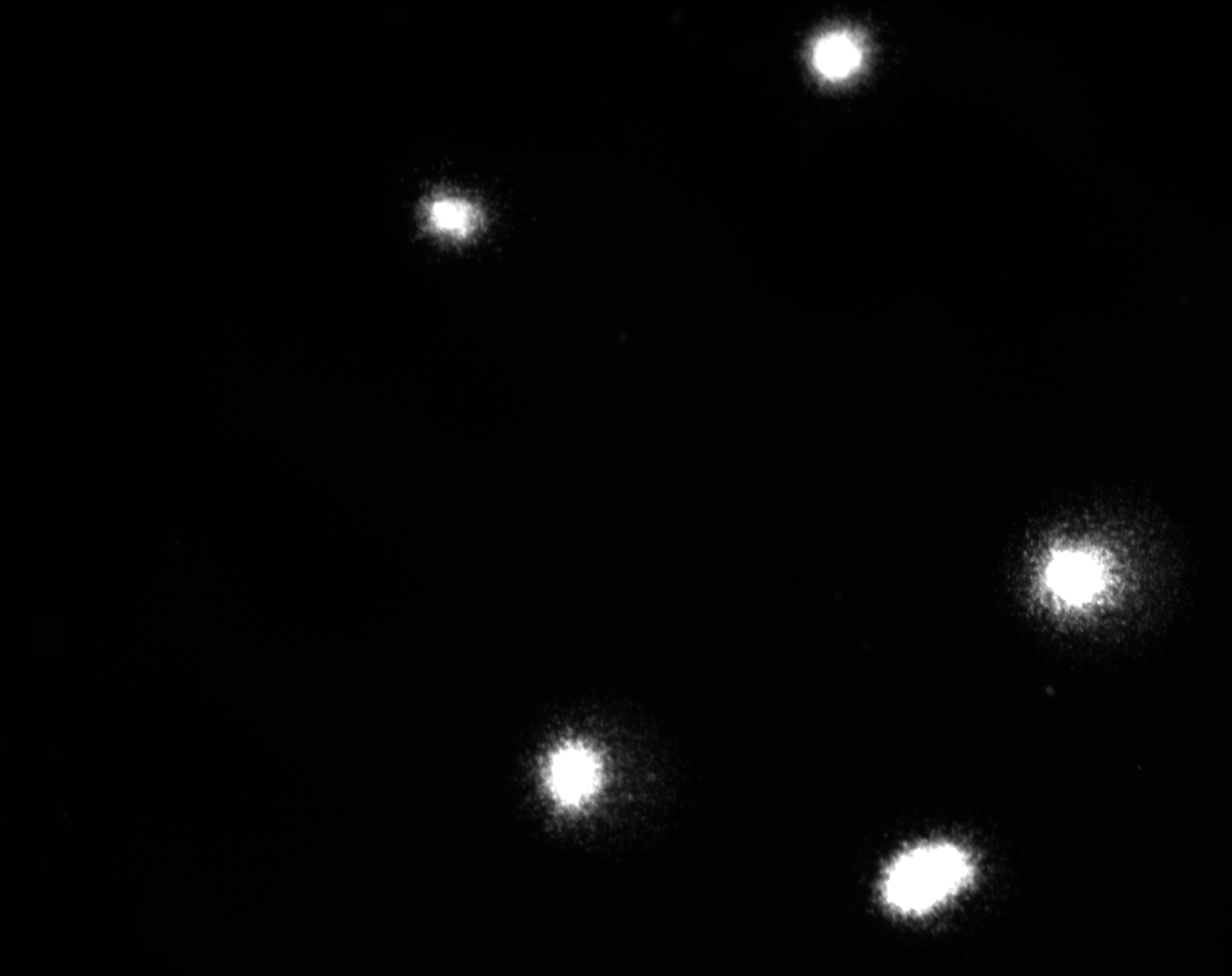


(**a**) (**b**) (**c**)

**Figure S3**. FACS report of cellular population in Annexin V assay. (**a**) C25, (**b**) T25, (**c**) E25+T25. For each image: Q3-1 represents the necrotic population, Q3-2 and Q3-4 represent the apoptotic population, Q3-3 represents the living population.

**
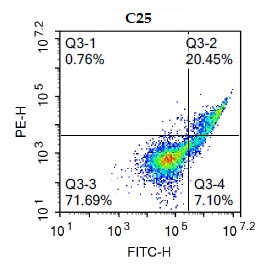

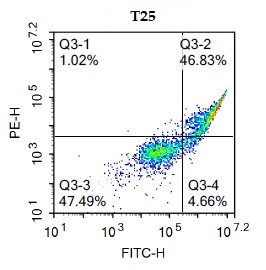

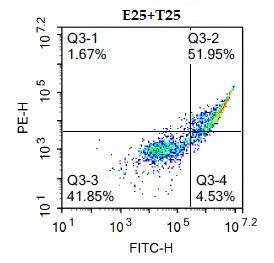
**

(**a**) (**b**) (**c**)
